# Prenatal and Newborn Immunoglobulin Levels from Mother-Child Pairs and Risk of Autism Spectrum Disorders

**DOI:** 10.3389/fnins.2016.00218

**Published:** 2016-05-18

**Authors:** Judith K. Grether, Paul Ashwood, Judy Van de Water, Robert H. Yolken, Meredith C. Anderson, Anthony R. Torres, Jonna B. Westover, Thayne Sweeten, Robin L. Hansen, Martin Kharrazi, Lisa A. Croen

**Affiliations:** ^1^California Department of Public HealthRichmond, CA, USA; ^2^Division of Research, Kaiser Permanente of Northern CaliforniaOakland, CA, USA; ^3^Department of Medical Microbiology and Immunology, University of CaliforniaDavis, CA, USA; ^4^Department of Internal Medicine, University of CaliforniaDavis, CA, USA; ^5^Johns Hopkins School of Medicine, Johns Hopkins UniversityBaltimore, MD, USA; ^6^Center for Persons with Disabilities, Utah State UniversityLogan, UT, USA; ^7^Department of Biology, Utah State UniversityBrigham City, UT, USA; ^8^MIND Institute, University of CaliforniaDavis, CA, USA; ^9^Genetic Disease Screening Program, California Department of Public HealthRichmond, CA, USA

**Keywords:** autism, maternal infection, biomarkers, immune function

## Abstract

**Background:** An etiological role for immune factors operating during early brain development in children with autism spectrum disorders (ASD) has not yet been established. A major obstacle has been the lack of early biologic specimens that can be linked to later diagnosis. In a prior study, we found lower risk of ASD associated with higher levels of maternally-derived total IgG and *Toxoplasmosis gondii* (Toxo) IgG in newborn blood spot specimens from children later diagnosed with ASD compared to population controls.

**Methods:** We obtained maternal mid-gestational serum specimens and newborn screening blood spots from the California Genetics Disease Screening Program (GDSP) for linked mother-baby pairs for 84 children with ASD and 49 children with developmental delay but not ASD (DD) identified from California Department of Developmental Services records and for 159 population controls sampled from birth certificates.Immunoglobulin levels in maternal and newborn specimens were measured by solid phase immunoassays and analyzed in logistic regression models for total IgG, total IgM, and Toxo IgG, and, for maternal specimens only, Toxo IgM. Correlations between maternal and newborn ranked values were evaluated.

**Results:** In both maternal and newborn specimens, we found significantly lower risk of ASD associated with higher levels of Toxo IgG. In addition, point estimates for all comparisons were < 1.0 suggesting an overall pattern of lower immunoglobulin levels associated with higher ASD risk but most did not reach statistical significance. We did not find differences in maternal or newborn specimens comparing children with DD to controls.

**Discussion:** These results are consistent with evidence from our prior study and other published reports indicating that immune factors during early neurodevelopment may be etiologically relevant to ASD. Lowered immunoglobulin levels may represent suboptimal function of the maternal immune system or reduced maternal exposure to common infectious agents.

**Conclusion:** Patterns seen in these selected immunoglobulins may provide clues to mechanisms of early abnormalities in neurodevelopment contributing to ASD. We recommend further study of immunoglobulin profiles in larger samples of linked mother-baby pairs to evaluate possible etiologic relevance.

## Introduction

Whether and how immune factors operating during fetal brain development are etiologically relevant to autism spectrum disorders (ASD) has not yet been determined. Numerous studies have documented atypical immune findings in some individuals already diagnosed with ASD. These include post-mortem brain tissue studies demonstrating evidence of chronic CNS inflammation in individuals aged 4–40 years old with ASD (Goines and Van de Water, 2010; Onore et al., 2012). More directly implicating a prenatal etiologic pathway, animal model studies have shown that autism-relevant behaviors can be induced in offspring following in utero exposure to antibodies derived from mothers with a child already diagnosed with ASD (Martin et al., 2008; Singer et al., 2009; Braunschweig et al., 2012), but not prenatal exposure to antibodies derived from serum of children with ASD (Morris et al., 2009). Other animal studies provide evidence that activation of the maternal immune system during pregnancy, either through infectious or non-infectious agents, can lead to atypical behaviors similar to those in ASD-affected children (Patterson, 2011). These findings are consistent with observational human studies that report maternal viral exposure (Chess, 1971; Deykin and MacMahon, 1979; Markowitz, 1983; Stubbs et al., 1984; Yamashita et al., 2003; Atladottir et al., 2012a; Zerbo et al., 2013) or inflammation (Brown et al., 2014) during pregnancy to be associated with increased risk of ASD in some children. Evidence for an association between prenatal infection and risk of other psychiatric disorders, such as schizophrenia and bipolar disorder, has also been reported (Buka et al., 2001a, 2008; Brown, 2011; Mortensen et al., 2011; Kneeland and Fatemi, 2013).

Despite this growing and complex body of research, questions regarding an etiologic role for immune factors in ASD remain to be resolved, impeded, in part, by a paucity of biologic specimens obtained during the presumed vulnerable period of fetal brain development and linked to later behaviorally-based diagnostic information. Routinely collected and archived specimens in California provide a resource for addressing some of these questions.

Using archived maternal specimens from California originally obtained during routine prenatal screening (Early Markers for Autism Study/EMA), Croen et al. (2008a) found differences in maternal mid-gestation antibody reactivity to human fetal brain protein in specimens from mothers of children with ASD compared to population-based controls. Although, the differences did not reach statistical significance, they are generally consistent with those based on maternal specimens obtained after an ASD diagnosis of an affected child (Dalton et al., 2003; Zimmerman et al., 2007; Braunschweig et al., 2008). Using the same prenatal specimens from the EMA project, Goines et al. (2011) reported a pro-inflammatory cytokine profile in mothers of children with ASD that was different from that of mothers of children without ASD.

In another California study, we measured neonatal levels of selected IgG, IgM, and IgA antibodies in archived newborn screening blood specimens to evaluate the hypothesis that higher antibody levels would be associated with a risk of ASD (Grether et al., 2010). In newborns, IgG antibodies are largely derived from the mother and transferred across the placenta to the fetus (Simister, 2003). Neonatal IgM and IgA antibodies are generally made by the fetus or neonate and serve as one set of putative markers of perinatal infection. In this earlier California study (Grether et al., 2010) in which we evaluated total IgG, IgM, and IgA as well as common viral antigen- specific immunoglobulins, we found no evidence of elevated immunoglobulin levels that would indicate increased prenatal infectious exposure in children with ASD, but we could not confidently rule out a role for all possible antigens or early transient exposures. Contrary to our hypothesis and of uncertain clinical significance, we found significantly lower ASD risk associated with higher levels of total IgG and *Toxoplasmosis gondii* (Toxo) IgG in the newborn specimens from the children with ASD. If supported by further research, these results may plausibly represent suboptimal humoral function in the maternal immune system and/or impaired transplacental transfer; the findings may also represent a protective effect in the controls in the postnatal period from earlier maternal exposure. As maternal specimens were not available for the children in that study, we were unable to further explore possible pathways.

We here report a case-control study using maternal mid-gestational as well as neonatal specimens from linked mother-child pairs to re-evaluate total IgG, total IgM, and *Toxoplasma gondii* immunoglobulins in children with ASD compared to population-based controls. To assist with interpretation of results, we also included a group of children with other developmental disabilities, comparing their assay results to control values. The study sample comes from the Early Markers for Autism (EMA) Study, a population-based, nested case-control study designed to evaluate biologic markers of susceptibility and exposure in archived maternal mid-gestational and neonatal blood specimens from linked mother-baby pairs.

## Methods

### Subjects

Study subjects were selected from the population of offspring born to women living in Orange County, California who were pregnant in 2000–2001 and enrolled in the State's Prenatal Expanded Alphafetoprotein Screening Program (Croen et al., 2008a) and for which specimens were available in the Project Baby's Breath (PBB) special research archive (see below). Three groups of children were identified: children with ASD, children with other developmental delay but not ASD (DD), and general population controls. Children with ASD or DD represent all children identified with these conditions who met the above criteria and who were enrolled with the Regional Center of Orange County (RCOC), one of 21 Regional Centers operated by the California Department of Developmental Services (DDS) to coordinate services for persons with ASD, developmental delay, and other developmental disabilities. Possible ASD cases were initially ascertained as clients receiving DDS services for autistic disorder or clients receiving services for other DDS-eligible conditions but who also had a code indicating ASD in the electronic record. Possible DD cases were initially ascertained as DDS clients without any evidence of ASD in the electronic records and with evidence of intellectual disability.

To confirm ASD or DD case status, we followed a protocol initially developed by the Metropolitan Atlanta Developmental Disabilities Surveillance Program (Autism and Developmental Disabilities Monitoring Network Surveillance Year 2006 Principal Investigators; Centers for Disease Control and Prevention (CDC), 2009), employing trained medical record abstractors to compile detailed diagnostic and clinical data from RCOC records for all children ascertained as possible ASD or possible DD. A pediatrician with certification in developmental and behavioral pediatrics (RH) then conducted expert clinical review of abstracted data to confirm ASD or DD case status using DSM IV criteria. Because of etiologic questions regarding co-morbid intellectual disability in children with ASD, children with ASD were further categorized by presence or absence of intellectual disability using DSM-IV criteria and based on standardized cognitive and adaptive assessments. Children with all scores < 70 were coded as having ID; children with all scores ≥70 or some scores < 70 and others ≥70 were coded as not having ID; children with no standardized scores in their chart were coded as “unknown.”

Controls were randomly sampled from the birth certificate files, frequency matched to ASD cases by sex, birth month, and birth year in a 2:1 ratio; all past or current DDS clients were identified through statewide electronic files and excluded from the control population. Demographic variables for all subjects were obtained from live birth certificates.

### Specimen collection

Maternal mid-gestational specimens were retrieved from the Project Baby's Breath (PBB) prenatal screening specimen archive maintained by the California Genetic Disease Screening Program (GDSP), California Department of Public Health. Following the completion of routine prenatal screening conducted by GDSP, PBB had retained any remaining portions of specimens in three selected southern California counties and selected birth years for analysis in approved research studies. During the study period, prenatal screening was conducted for approximately 70% of pregnancies in the state (state law mandates that all women in the first half of pregnancy be offered the voluntary screening). Venous blood was collected at 15–20 weeks gestation in serum separator tubes by obstetrical care service providers and laboratories, and underwent expanded alpha fetoprotein (XAFP) screening at a single regional laboratory, typically within 7 days of collection (median time = 3 days). During transit via US Postal Service to the regional screening laboratory, no effort was made to control the temperature of the specimens. After testing, remaining portions of specimens were kept under refrigeration for 1–2 days and then stored at −20°C by the regional laboratory and PBB. Aliquots of the samples used for this study were shipped to the laboratory of Dr. Judy van de Water, UC Davis, and stored at −80°C until use with a single thaw prior to immunoglobulin testing. All samples were exposed to the same collection and storage protocols.

Newborn screening filter paper blood specimens were obtained from the newborn archive maintained by GDSP that conducts routine metabolic screening for all newborns in the state. Specimens from cases and controls were located, one spot bar-coded with a study identifier, and shipped on dry ice, without regard to case-control status, to The Center for Persons with Disabilities (CPD) at Utah State University. Working under aseptic conditions, researchers in the CPD lab prepared 96-well plates, each well containing two 3 mm diameter punches from one archived specimen. Since original blood draw and newborn screening, specimens have been stored at −20°C.

All prenatal and newborn specimens were then shipped on dry ice to the Neurovirology Laboratory, Johns Hopkins School of Medicine, for analysis, blinded to case-control status, using enzyme immunoassays as described below.

### Laboratory assays

We measured antibodies in both maternal and newborn specimens by solid phase immunoassays (ELISA). Maternal and newborn total IgG and total IgM levels were expressed as μg/ml. In maternal specimens, we also measured *Toxoplasma gondii* (Toxo) IgG with resulting levels expressed as standardized international units (SI) and Toxo IgM with resulting levels expressed as blank-adjusted optical density units. In newborn specimens, we measured Toxo IgG with resulting levels expressed as blank-adjusted optical density units. Assay results were not corrected for hematocrit or total protein and specimens lacking detectable signals for a specific assay were considered at the minimum detection level (MDL) for that assay.

### Exclusions

Subjects (1 child with ASD, 3 children with DD, 1 control child) were excluded due to estimated gestational age < 26.0 completed weeks (182 days; recorded on the birth certificate and based on LMP) or because age at blood draw was >7.0 days after birth according to the newborn screening record.

### Statistical methods

To take into consideration assay detection limits, unknown distributions of the immunoglobulins, and inadequate knowledge of the biologic significance of immunoglobulin levels measured in archived specimens, we conducted primary analyses treating the measured immunoglobulin values as categorical variables. Categories were constructed separately for each of the analytes based on percent of signals at the MDL for these assays: if ≤ 25% of controls were at the MDL, observations were divided into quartiles based on control values, with MDL values included in lowest quartile (Q1); if >25% of controls had values at the MDL, then MDL values were designated as the lowest category (CAT1) and remaining values were divided into three equal-sized categories based on control values. The exception to this was newborn total IgM, which had only a low and a high category due to limited distribution.

For comparisons between ASD and controls, and separately between DD and controls, we then conducted crude and adjusted analyses using logistic regression to estimate the risk of ASD (or DD) associated with each analyte separately, comparing the lowest category to each higher category using odds ratios (ORs) considered statistically significant if 95% confidence intervals did not include 1.0. Adjusted models included assay plate (5 maternal and 5 newborn) as additional independent variables and covariates associated with ASD (or DD) in these data (*p* < 0.5; Table 1). Due to small numbers of children with DD, we collapsed the three higher quartiles (or categories) into one group (HIGH) and used the lowest quartile (or category) as the reference group (LOW). We explored differences in immunoglobulin levels between children with ASD with ID and those with ASD without ID, comparing each of these subgroups to the control group. Small numbers of subjects in subgroups precluded the use of adjusted models.

**Table 1:** **Demographic characteristics of children with Autism Spectrum Disorders (ASD), developmental delay without ASD (DD), and Control Subjects. Early Markers for Autism (EMA) Study, births 2000, 2001**.

	**Controls (*****N*** = 159**)**		**Children with ASD (*****N*** = 84**)**			**Children with DD (*****N*** = 49**)**		
**Baby characteristics**	***N***	**%**	***N***	**%**	***P*-value^*a*^**	***N***	**%**	***P*-value^*a*^**
**GENDER**								
Male	139	87.42	73	86.90	0.91	29	59.18	< 0.0001
Female	20	12.58	11	13.10		20	40.82	
**BIRTH YEAR**								
2000	31	19.5	23	27.38	0.16	26	53.06	< 0.0001
2001	128	80.5	61	72.62		23	46.94	
Birth Month					0.96			0.01
**PLURALITY**								
Singleton	156	98.11	81	96.43	0.42	47	95.92	0.34
Multiple	3.00	1.81	3	3.57		2	4.08	
	**Mean**	**(SD)**	**Mean**	**(SD)**	***P*****-value**^b^	**Mean**	**(SD)**	***P*****-value**^b^
Gestational age at birth (days)	271.01	(14.27)	272.12	(18.47)	0.63	266.33	(28.12)	0.27
Birth weight	3348.1	(616.09)	3416.1	(699.1)	0.44	2897.7	(827.22)	0.0008
Days between birth and blood draw	1.46	(0.94)	1.48	(0.71)	0.80	1.92	(1.43)	0.036
Protein concentration in newborn specimen (ug/ml)	6520.3	(1377.2)	6495.5	(1222.1)	0.89	6404.4	(1551.5)	0.62
**Maternal characteristics**	***N***	**%**	***N***	**%**	***P*****-value**^a^	***N***	**%**	***P*****-value**^*a*^
**MATERNAL RACE/ETHNICITY**								
White non-Hispanic	53	33.33	37	44.05	0.0005	9	18.37	*0.43*
White Hispanic: US Born	13	8.18	10	11.9		5	10.20	
White Hispanic: Not US Born	60	37.74	10	11.9		23	46.94	
Black	1	0.63	0	0.00		1	2.04	
Asian	28	17.61	19	22.62		9	18.37	
Other	4	2.52	8	9.52		2	4.08	
**MATERNAL AGE**								
< 20	9	5.66	2	2.38	0.004	3	6.12	0.90
20–24	33	20.75	7	8.33		8	16.33	
25–29	48	30.19	18	21.43		18	36.73	
30–34	51	32.08	38	45.24		14	28.57	
35+	18	11.32	19	22.62		6	12.24	
**MATERNAL EDUCATION**								
< High school	49	30.82	7	8.43	< 0.0001	22	44.90	0.23
High school graduate	39	24.53	15	18.07		9	18.37	
College	46	28.93	40	48.19		14	28.57	
Post graduate	25	15.72	21	25.3		4	8.16	
**PARITY**								
Primiparous	67	42.14	42	50.00	0.24	16	37.65	0.24
Multiparous	92	59.86	42	50.00		33	67.35	
**INTER-LIVEBIRTH INTERVAL (MONTHS)**								
0–12	16	17.58	8	20.00	0.68	4	12.50	0.52
13–24	19	20.88	10	25.00		10	31.25	
25–60	31	34.07	15	37.50		12	37.50	
>60	25	27.47	7	17.50		6	18.75	
	**Mean**	**(SD)**	**Mean**	**(SD)**	***P*****-value**^b^	**Mean**	**(SD)**	***P*****-value**^b^
Gestational age at blood draw (days)	118.78		119.76		0.35	118.37		0.75
Mother's weight at XAFP blood draw (lbs)	146.89	(33.78)	145.07	(26.72)	0.65	150.3	(39.3)	0.56

a *Chi-square test of association, comparing to controls*.

b *t-test for equality of means, comparing to controls*.

To evaluate the association between maternal mid-gestational analyte signals and those detected in their children's newborn specimens, we computed Spearman rank-order correlation coefficients separately for cases and controls for the three analytes measured in both mothers and newborns (total IgG, total IgM, and Toxo IgG). Values at the MDL were treated as the lowest category, with measured signals above the MDL for each individual treated as categorical variables in the computations.

### Institutional review board

This study was approved by the California Health and Human Services Agency Committee for the Protection of Human Subjects (IRB) and the Kaiser Permanente of Northern California Institutional Review Board, who granted a waiver of informed consent. The analytic laboratories operated under an IRB exemption as the samples were preexisting and did not have personal identifiers.

## Results

The final study group included 84 children with ASD, 49 children with DD, and 159 controls. No significant differences were found between ASD and controls on the matching variables (gender, birth month, birth year), nor for single or multiple birth, gestational age (GA), birth weight, days between birth and blood draw, and protein concentration in the newborn specimen (Table 1). Mothers of children with ASD were similar to controls with regard to parity, inter-pregnancy interval (number of months between birth of index child and prior live birth), GA at mid-gestational blood draw, and maternal weight at blood draw (Table 1). ASD cases were more likely than controls to have mothers who self-identified as white non-Hispanic, were older, and more educated.

Compared to the control children, children with DD were less likely to be male and born in the first study year (2000) or during the same birth months due to matching of the controls with the ASD group on these variables. Children with DD were also of lower birth weight and had a longer time between birth and blood draw than controls (Table 1). No differences in maternal characteristics were observed.

For the ASD study group, both newborn and maternal specimens were available for 80 mother-baby pairs and only maternal specimens were available for the remaining 4 mother-baby pairs; for the DD study group, both specimens were available for 45 pairs, only maternal specimen for 1 pair, and only newborn specimens for 3 pairs; for the controls, both specimens were available for 141 pairs, only maternal specimens for 6 pairs, and only newborn specimens for 12 pairs. Signals above the MDL were detected for total IgG and total IgM in all maternal and newborn specimens and for Toxo IgM in all maternal specimens. For the Toxo IgG assay, 93 maternal specimens were at the MDL (45% of ASD, 33% of DD, 27% of controls), as was one newborn specimen for a child with DD.

### ASD vs. controls

The distributions of each analyte for ASD cases and controls are shown in Table 2. In unadjusted analyses, measured levels of total IgG, total IgM, and Toxo IgM in mid-gestational specimens of mothers of children diagnosed with ASD were not significantly different from population control values but virtually all point estimates were substantially below 1.0 (Table 2). For Toxo IgG, significantly fewer mothers of children with ASD were represented in CAT3 and CAT4 than were mothers of controls (Table 2), indicating lower risk of ASD with higher measured antibody.

**Table 2:** **Association of antibodies and risk of ASD, distribution and crude odds ratios, EMA study, births 2000–2001**.

	**N at MDL**	**Category**	**Analyte**	**ASD**	**Controls**	**OR_*unadj*_**	**95% CI**
**MATERNAL**							
Total IgG (μg/ml)		Q4	>53.63	18	36	0.77	(0.36, 1.66)
		Q3	>46.59–53.63	28	37	1.17	(0.57, 2.37)
		Q2	>43.11–46.59	14	37	0.58	(0.26, 1.30)
	0	(Ref^*^) Q1	0–43.11	24	37		
Total IgM (μg/ml)		Q4	>65.96	15	35	0.55	(0.25, 1.19)
		Q3	>56.80–65.96	18	38	0.60	(0.29, 1.27)
		Q2	>29.56–56.80	22	37	0.76	(0.37, 1.56)
	0	(Ref^*^) Q1	0–29.56	29	37		
Toxo IgG (SI)		Cat 4	>18.6	8	37	**0.23**	**(0.09, 0.55)**
		Cat 3	>6.3–18.6	15	35	**0.45**	**(0.21, 0.96)**
		Cat 2	>0–6.3	23	35	0.69	(0.35, 1.38)
	78	(Ref^**^) Cat 1	0	38	40		
Toxo IgM (odu^***^)		Q4	>0.202	15	35	0.57	(0.26, 1.23)
		Q3	>0.172–0.202	22	38	0.77	(0.38, 1.56)
		Q2	>0.158–0.172	17	34	0.67	(0.32, 1.41)
	0	(Ref^*^) Q1	0–0.158	30	40		
**NEWBORN**							
Total IgG (μg/ml)		Q4	>61.18	18	38	0.66	(0.31, 1.38)
		Q3	>55.03–61.18	16	38	0.59	(0.27, 1.25)
		Q2	>51.63–55.03	18	38	0.66	(0.31, 1.38)
	0	(Ref^*^) Q1	0–51.63	28	39		
Total IgM (μg/ml)		Cat2	>1.20	29	66	0.75	(0.43, 1.31)
	0	(Ref^**^) Cat 1	0–1.20	51	87		
Toxo IgG (odu^***^)		Q4	>0.097	5	38	**0.14**	**(0.05, 0.39)**
		Q3	>0.066–0.097	23	38	0.64	(0.32, 1.27)
		Q2	>0.057–0.066	15	38	**0.42**	**(0.20, 0.88)**
	0	(Ref^*^) Q1	0–0.057	37	39		

* *Reference quartile (Q1) includes signals at and above the MDL based on control values*.

** *Reference category (Cat 1) only includes signals at the MDL. Values above the MDL divided into equal-sized categories based on control values*.

*** *Optical density units (blank adjusted)*.

*Bold means statistically significant*.

In unadjusted analyses, newborn specimens from children with ASD did not show significantly different levels from controls for total IgG or total IgM. For Toxo IgG, risk was reduced for signals above Q1, but only significantly so for Q2 and Q4 (Table 2). All ORs were below 1.0 in the newborn comparisons.

After adjustment for laboratory plate, maternal age, and maternal education (Table 3_Adj Column 1_), maternal specimens did not show significant difference between cases and controls for total IgG, total IgM, or Toxo IgM, although, as in the unadjusted analyses, virtually all point estimates were below 1.0. Higher levels of Toxo IgG were significantly associated with lower risk of ASD (Category 4 vs. Category 1) and the dose-response trend across the Toxo IgG categories reached statistical significance (*p* = 0.038). With further adjustment for maternal race-ethnicity (Table 3_Adj Column 2_), the Toxo IgG results continued to indicate lower risk with higher levels with a dose-response pattern (*p* = 0.039). Further adjustment that included place of birth for white Hispanic mothers (US-born or not US-born) showed similar patterns that did not reach statistical significance (Table 3_Adj Column 3_).

**Table 3:** **Association of antibodies and risk of ASD, EMA study, births 2000–2001**.

	**OR${}_{\text{adj}}^{\text{a}}$**	**95% CI**	**OR${}_{\text{adj}}^{\text{b}}$**	**95% CI**	**OR${}_{\text{adj}}^{\text{c}}$**	**95% CI**
**MATERNAL**						
Total IgG						
Q4 vs. Q1	0.92	(0.39, 2.17)	0.87	(0.37, 2.08)	0.89	(0.37, 2.13)
Q3 vs. Q1	1.41	(0.63, 3.14)	1.39	(0.62, 3.12)	1.34	(0.59, 3.01)
Q2 vs. Q1	0.53	(0.22, 1.30)	0.51	(0.21, 1.25)	0.51	(0.21, 1.27)
Total IgM						
Q4 vs. Q1	0.46	(0.17, 1.27)	0.44	(0.16, 1.23)	0.42	(0.15, 1.21)
Q3 vs. Q1	0.63	(0.25, 1.61)	0.63	(0.25, 1.60)	0.71	(0.27, 1.85)
Q2 vs. Q1	0.68	(0.29, 1.62)	0.69	(0.29, 1.66)	0.75	(0.31, 1.82)
Toxo IgG						
CAT4 vs. CAT1^*^	**0.35**	**(0.13, 0.91)**	**0.33**	**(0.12, 0.86)**	0.40	(0.15, 1.11)
CAT3 vs. CAT1^*^	0.58	(0.25, 1.32)	0.53	(0.22, 1.24)	0.55	(0.23, 1.30)
CAT2 vs. CAT1^*^	0.86	(0.40, 1.86)	0.83	(0.38, 1.81)	0.86	(0.39, 1.87)
Toxo IgM						
Q4 vs. Q1	0.80	(0.32, 1.99)	0.78	(0.31, 1.94)	0.71	(0.28, 1.77)
Q3 vs. Q1	0.82	(0.36, 1.84)	0.78	(0.34, 1.78)	0.70	(0.30, 1.64)
Q2 vs. Q1	0.78	(0.33, 1.84)	0.74	(0.31, 1.78)	0.69	(0.28, 1.69)
**NEWBORN**						
Total IgG						
Q4 vs. Q1	0.77	(0.31, 1.89)	0.76	(0.31, 1.86)	0.70	(0.28, 1.76)
Q3 vs. Q1	0.65	(0.28, 1.48)	0.61	(0.27, 1.42)	0.64	(0.27, 1.48)
Q2 vs. Q1	0.75	(0.33, 1.69)	0.72	(0.32, 1.65)	0.72	(0.31, 1.66)
Total IgM						
HIGH vs. LOW^**^	0.67	(0.30, 1.49)	0.67	(0.30, 1.48)	0.77	(0.34, 1.75)
Toxo IgG						
Q4 vs. Q1	**0.25**	**(0.08, 0.79)**	**0.25**	**(0.08, 0.78)**	0.31	(0.10, 1.01)
Q3 vs. Q1	0.89	(0.41, 1.94)	0.90	(0.41, 1.96)	0.87	(0.39, 1.92)
Q2 vs. Q1	0.52	(0.23, 1.17)	0.52	(0.23, 1.18)	0.50	(0.22, 1.14)

*^a^Adjusted for laboratory plate, maternal age, maternal education*.

*^b^Adjusted for laboratory plate, maternal age, maternal education, maternal race/ethnicity (White non-Hisp., White Hisp., Other)*.

*^c^Adjusted for laboratory plate, maternal age, maternal education, maternal race/ethnicity (White non-Hisp., White Hisp.-US Born, White Hisp.-Not US Born, Other)*.

* *Reference category (CAT1) only includes signals at the MDL. Values above the MDL divided into 3 equal-sized categories based on control values*.

** *Reference category (LOW) includes all signals above but close to the minimum observed value for the assay, with remaining values lumped into one higher category*.

*Bold means statistically significant*.

In adjusted models with newborn specimens, all ORs were below 1.0, with the comparison of Toxo IgG Q4 vs. Q1 reaching statistical significance (Table 3_Adj Columns 1 and 2_). No dose-response pattern for newborn Toxo IgG was observed.

### ASD with and without intellectual disability

Data on presence/absence of intellectual disability was available on 76% of the children with ASD, permitting identification of two subgroups: ASD with ID (*n* = 34) and ASD without ID (*n* = 30). Each of these subgroups was then compared separately to the control group and the resulting ORs for each analyte compared across the two subgroups. No statistically significant differences between the two subgroups were observed (data not shown). Small numbers of children precluded adjustment for covariates.

### DD vs. controls

In models adjusted for laboratory plate, gender, birth year, and days to blood draw, we found no association between risk of DD and any of the analytes when comparing the LOW and HIGH exposure categories. Odds ratios for the different analytes ranged from 0.71 to 1.28 with no apparent pattern and all confidence intervals included the null value (data not shown).

### Correlations within mother-baby pairs

Within mother-baby pairs, levels of total IgG measured in mothers during mid-gestation and in their newborns were not correlated in the group of ASD cases (*r*_φ_ = −0.03), in controls (*r*_φ_ = 0.04), or in the DD group (*r*_φ_ = 0.08). Similarly, IgM showed no correlation between levels in maternal and newborn specimens in the ASD group (*r*_φ_ =0.04), the control group (*r*_φ_ = −0.03) or the DD group (*r*_φ_ = −0.0.26, *p* = 0.08). In contrast, Toxo IgG levels in maternal and newborn specimens were correlated within ASD cases (*r*_φ_ = 0.34, *p* < 0.002) and within population controls (*r*_φ_ = 0.53, *p* < 0001) but not in the DD group (*r*_φ_ = 0.11, *p* = 0.47).

To further explore an association between low Toxo IgG and ASD, we evaluated mother-baby ASD and control pairs in which both mother and newborn Toxo IgG levels were in the reference range (see Table 2) compared to pairs in which either or both the maternal or the newborn values were above the reference range. The logistic model (adjusted for laboratory plate only) yielded an OR of 0.24 (95% CI 0.10, 0.56), consistent with a lower risk of ASD associated with higher levels of Toxo IgG.

## Discussion

This ASD case-control study is the first to report immunoglobulin levels measured in both maternal mid-gestational and newborn specimens from linked mother-baby pairs. The immunoglobulins were selected based on our prior California study using newborn specimens in which lower risk of ASD was associated with higher assay levels for total IgG and Toxo IgG (Grether et al., 2010).

The results reported here are consistent in many, but not all, respects to those we reported earlier. In the current study, the results for both total IgG and Toxo IgG suggest, but do not statistically confirm, a lower risk of ASD associated with higher levels of the analytes in newborn specimens, similar to the earlier findings but not reaching statistical significance, perhaps because of the smaller number of subjects available for this current study. In the maternal specimens, lower risk of ASD appeared to be associated with higher measured levels of Toxo IgG, reaching statistical significance for several comparisons with a dose-response pattern indicating a protective association. However, this association no longer reached statistical significance in models controlling for maternal place of birth, suggesting that the ASD case and controls differences we see with Toxo IgG may be a function of place of birth, as country of origin is a significant factor influencing exposure to Toxo (Jones et al., 2001).

The ORs for most comparisons, both maternal and newborn, were substantially below the null value, suggesting an overall pattern of lower measured levels of these immunoglobulins in ASD mother-baby pairs, even though some risk estimates did not reach statistical significance. Whether these patterns would become more statistically robust with a larger number of subjects cannot be determined from this one study. No differences were detected in immunoglobulin levels between subgroups of ASD defined by presence/ absence of intellectual disability, but sample sizes were small. Analyses of the analytes in specimens from a heterogeneous group of children with DD and their mothers failed to show any associations or possible patterns when compared to the controls, and small numbers of children with DD prevented detailed analysis.

Within mother-baby pairs, the levels of total IgG for mothers and their offspring were not statistically correlated for any of the three groups of subjects; nor were mother-baby values correlated for total IgM. Absence of correlation is, perhaps, not surprising given the elapsed time between maternal mid-gestational and newborn blood draws, the limitation of one time-point during pregnancy, and the multitude of antigens and immune response represented in these total measurements (Buka et al., 2001b). In contrast, signals for antigen-specific Toxo IgG show moderate and statistically significant correlation between maternal mid-gestational and newborn values for both ASD and population control pairs, despite the limitations related to time of specimen collection. Within the context of this study, we are unable to determine if the presence of Toxo IgG represents recent or distant exposure (Toxo IgG can persist for several years), but to our knowledge, none of the children in either the ASD or population control groups showed clinical manifestations of congenital Toxoplasmosis. For the DD group, no correlations were observed within mother-baby pairs for any analytes, perhaps because of the greater number of children of low gestational age in this subgroup (transfer of IgG antibodies from mother to baby occurs late in gestation).

Because this study employs different specimens and assays than those used in clinical settings, the clinical significance of the differences observed here is unclear. However, the overall consistency of our results with those found in our prior study (Grether et al., 2010), of higher ASD risk associated with lower immunoglobulin signals, is potentially informative. Taken together, these results are consistent with the evidence from other studies indicating that atypical maternal immune function during fetal life may contribute in some way to the development of ASD in a subset of children (Croen et al., 2008a; Goines et al., 2011; Brown et al., 2014).

In the prior study, we raised the question of whether the higher risk of ASD associated with lower newborn immunoglobulin signals might represent impaired placental transport. Based on the current study, this does not seem likely since the ASD maternal specimens showed the same overall pattern of lower immunoglobulin levels compared to controls as seen in the newborn specimens.

A plausible explanation for lower immunoglobulin levels associated with ASD is suboptimal function of the maternal immune system; exposure to common antigens may not elicit a full or typical maternal immune response, contributing to development of ASD in offspring. Whether and how suboptimal maternal immune function may impact fetal neurodevelopment remains to be explicated. Another possible explanation is that the lower immunoglobulin levels associated with ASD may simply represent reduced maternal exposure to common infectious agents. That some version of the “hygiene hypothesis” during early neurodevelopment may be relevant to ASD risk was previously offered by Becker (2007) based on parallel epidemiological, morphometric, molecular, and genetic patterns between ASD and other inflammatory disorders such as asthma.

Infants and young children who lack the robust immune protection provided by maternally-derived immunoglobulins, whether from suboptimal maternal immune function or reduced maternal exposure, might be expected to be more vulnerable to infectious exposures after birth, perhaps contributing to higher risk for ASD. However, studies have failed to consistently show an association between increased frequency of postnatal infectious illnesses and risk for ASD (Rosen et al., 2007; Atladottir et al., 2010, 2012b). Whether postnatal exposure to *Toxoplasmosis* as a specific antigen may be related to higher risk for ASD has not, to our knowledge, been investigated.

Animal studies have documented a link between maternal infectious exposure (and non-infectious activation of the maternal immune system) during pregnancy and autistic-like behaviors in offspring (Patterson, 2011). A number of early human studies also indicated that maternal infectious illnesses during pregnancy, although uncommon, may be more frequent in the history of children with ASD than in controls (Chess, 1971; Deykin and MacMahon, 1979; Markowitz, 1983; Yamashita et al., 2003). More recent studies are inconsistent (Atladottir et al., 2012a; Zerbo et al., 2013; Brown et al., 2014). Further studies based on medical record documentation may be informative, at least for more severe infections/fevers, as would prospective studies that document maternal illness during pregnancy.

In interpreting the results of our study, several strengths and limitations need to be considered. Strengths include population-based identification of subjects and the prospective specimen collection, permitting analysis of biological markers of infections during early fetal and neonatal neurodevelopment. However, our modest sample size limited our statistical power, especially for DD group for which our null results may not be generalizable. The diagnostic information we used to select the ASD and DD groups relied on DDS service agency reports with expert review by experienced clinicians. Other studies, in which the ASD diagnosis has been validated through comprehensive clinical assessment, have indicated a very high level of diagnostic validity for children enrolled in DDS with an ASD (Hertz-Picciotto et al., 2006; Croen et al., 2008b; Hallmayer et al., 2011). Our laboratory assays were fully blinded to case-control status and conducted in research laboratories with extensive experience in these research assays. However, the assays are not comparable to clinical ones, limiting our ability to interpret the biologic relevance of the results in a clinical framework. Since we did not measure all antigen-specific immunoglobulins, our results should not be interpreted as ruling out the possibility that exposure to some specific antigens not evaluated here might increase risk of ASD. Bias related to laboratory procedures can reasonably be ruled out as an explanation for our overall patterns of lower antibody levels in the ASD group, but undetected bias in initial selection of subjects must be considered as a possible explanation.

## Conclusion

Taken as a whole, and if supported by further research, our results suggest that early immunoglobulin patterns in ASD mother-baby pairs may be etiologically informative; we recommend further studies that include prenatal and newborn specimens from linked mother-baby pairs to evaluate these immunoglobulins and other immune markers and possible effect modification associated with gene-pool factors or geographic differences in maternal exposure.

## Author contributions

JG provided direction to the conception and design of the study, acquisition of data, conducted analysis and interpretation of data, and prepared the manuscript. PA contributed to conception and design of the study, analysis and interpretation of data, and revision of the manuscript for important intellectual content. JV contributed to conception and design of the study, preparation of specimens, analysis and interpretation of data, and revision of the manuscript for important intellectual content. RY contributed PA contributed actively to conception and design of the study, analysis and interpretation of data, and revision of the manuscript for important intellectual content. MA contributed to conception and design of the study, analysis and interpretation of data, and revision of the manuscript for important intellectual content. AT contributed to conception and design of the study, preparation of specimens, analysis and interpretation of data, and revision of the manuscript for important intellectual content. JW contributed to preparation of specimens, analysis and interpretation of data, and revision of the manuscript for important intellectual content. TS contributed to preparation of specimens, analysis and interpretation of data, and revision of the manuscript for important intellectual content. RH contributed to conception and design of the study, acquisition of data, and revision of the manuscript for important intellectual content. MK contributed to conception and design of the study, acquisition of data, analysis and interpretation of data, and revision of the manuscript for important intellectual content. LC contributed to the conception and design of the study, acquisition of data, analysis and interpretation of data, and review and revision of the manuscript. All authors read and approved the final manuscript.

## Funding

Study funded by the National Institute of Environment Health Sciences, 5R01ES016669.

### Conflict of interest statement

The authors declare that the research was conducted in the absence of any commercial or financial relationships that could be construed as a potential conflict of interest.

## Abbreviations

ASD: autism spectrum disorders
CNS: central nervous system
DDS: California Department of Developmental Services
CAT: category
CPD: The Center for Persons with Disabilities (CPD)
DD: developmental delay
DSM IV: Diagnostic and Statistical Manual of Mental Disorders, 4th Edition
ELISA: enzyme-linked immunosorbent assay
EMA: Early Markers for Autism Study
GA: gestational age
GDSP: California Genetics Disease Screening Program
HIGH: the three higher quartiles (or categories)
IgA: Immunoglobulin A
IgG: Immunoglobulin G
IgM: Immunoglobulin M
IRB: institutional review board
LOW: lowest quartile (or category)
MDL: minimum detection level
OR: odds ratio
PBB: Project Baby's Breath
Q: quartile
RCOC: Regional Center of Orange County
SI: standardized international units
Toxo: Toxoplasmosis gondii
XAFP: expanded alpha fetoprotein.
